# *Fpr2*^−/−^ Mice Developed Exacerbated Alcohol-Associated Liver Disease

**DOI:** 10.3390/biology12050639

**Published:** 2023-04-23

**Authors:** Josiah E. Hardesty, Jeffrey B. Warner, Ying L. Song, Alison Floyd, Craig J. McClain, Dennis R. Warner, Irina A. Kirpich

**Affiliations:** 1 Division of Gastroenterology, Hepatology, and Nutrition, Department of Medicine, University of Louisville, Louisville, KY 40202, USA; 2 Department of Pharmacology and Toxicology, University of Louisville, Louisville, KY 40202, USA; 3 Robley Rex Veterans Medical Center, Louisville, KY 40202, USA; 4 University of Louisville Alcohol Center, University of Louisville, Louisville, KY 40202, USA; 5 University of Louisville Hepatobiology & Toxicology Center, University of Louisville, Louisville, KY 40202, USA; 6 Department of Microbiology and Immunology, University of Louisville, Louisville, KY 40202, USA

**Keywords:** alcohol-associated liver disease, FPR2, restorative MoMFs

## Abstract

**Simple Summary:**

Alcohol-associated liver disease (ALD) is a global healthcare problem, and the mechanisms of ALD development are not fully understood. In this study, we found that loss of formyl peptide receptor 2 (FPR2) worsened alcohol-induced injury and inflammation and altered liver regeneration. An exacerbation of ALD due to FPR2 gene knockout was associated with abnormal immune responses. This study demonstrated that FPR2 plays an important role in the pathogenesis of ALD.

**Abstract:**

Alcohol-associated liver disease (ALD) is the most common chronic liver disease and carries a significant healthcare burden. ALD has no long-term treatment options aside from abstinence, and the mechanisms that contribute to its pathogenesis are not fully understood. This study aimed to investigate the role of formyl peptide receptor 2 (FPR2), a receptor for immunomodulatory signals, in the pathogenesis of ALD. WT and *Fpr2*^−/−^ mice were exposed to chronic–binge ethanol administration and subsequently assessed for liver injury, inflammation, and markers of regeneration. The differentiation capacity of liver macrophages and the oxidative burst activity of neutrophils were also examined. Compared to WT, *Fpr2*^−/−^ mice developed more severe liver injury and inflammation and had compromised liver regeneration in response to ethanol administration. *Fpr2*^−/−^ mice had fewer hepatic monocyte-derived restorative macrophages, and neutrophils isolated from *Fpr2*^−/−^ mice had diminished oxidative burst capacity. *Fpr2*^−/−^ MoMF differentiation was restored when co-cultured with WT neutrophils. Loss of FPR2 led to exacerbated liver damage via multiple mechanisms, including abnormal immune responses, indicating the crucial role of FPR2 in ALD pathogenesis.

## 1. Introduction

Alcohol-associated liver disease (ALD) is the most common chronic liver disease and a significant contributor to the global healthcare burden [1]. The spectrum of ALD ranges from liver steatosis to steatohepatitis, fibrosis, and cirrhosis. Worldwide, 500,000 annual cirrhosis deaths can be attributed to chronic alcohol consumption [2]. However, there is currently no effective FDA-approved therapy to halt or reverse ALD in humans. The pathogenesis of ALD is complex, with multiple molecules, signaling pathways, and cell types involved. Several mechanisms have been identified in ALD development and progression, including increased intestinal permeability and gut-derived endotoxemia [3], immune system dysfunction [4], hepatocellular death [5], and inflammation [6], among others. Impaired liver regeneration due to a loss of hepatocyte proliferation following alcohol-induced damage has also been identified as a contributing factor to the pathogenesis of ALD [7,8]. The elucidation of new molecules and signaling pathways contributing to ethanol-induced liver injury and altered regeneration may lead to the development of novel therapeutics for this disease.

Formyl peptide receptor 2 (FPR2) is a G-protein-coupled receptor highly expressed in multiple immune cells such as neutrophils, macrophages, monocytes, and monocyte-derived macrophages [MoMFs] [9,10]. FPR2 interacts with various ligands, which elicit a plethora of effects [10]. One such ligand is bacteria-derived N-Formyl methionyl-leucyl-phenylalanine, which stimulates neutrophil chemotaxis in vitro [11]. FPR2 ligands that originate from the host, e.g., Annexin A1, have been shown to increase IL-10 production in monocytes [12]. Resolvin D1 is another host-derived FPR2 ligand that can promote M2 polarization of hepatic macrophages and increase efferocytosis capacity [13]. It has been shown that the loss of FPR2 can exacerbate the pathology of several diseases [13,14,15,16]. Previous studies demonstrated that *Fpr2*^−/−^ mice developed exacerbated liver injury and inflammation in animal models of sepsis [17] and increased mortality after bacterial infections [14]. In a diet-induced, non-alcohol-associated fatty liver disease model, *Fpr2^−/−^* mice also developed exacerbated liver injury and inflammation [15]. However, the role of FPR2 in ALD pathogenesis has not been investigated. In the current study, we aimed to examine whether *Fpr2* genetic deletion can exacerbate ethanol (EtOH)-induced liver injury in an animal model of acute-on-chronic EtOH administration and identify the contributing mechanisms.

## 2. Materials and Methods

### 2.1. Animal Studies

*Fpr2*^−/−^ mice on a C57BL/6J background were generated by Dr. Mauro Perretti [18] and obtained from Dr. Asma Nusrat (University of Michigan). Mice were housed in a temperature-controlled room (23.9 °C) with a 12 h light-dark cycle. Experimental *Fpr2^−/−^* mice were bred in-house and genotyped as detailed in [18]. qRT-PCR analysis confirmed that *Fpr2^−/−^* mice did not express the *Fpr2* transcript (Figure S1). female *Fpr2*^−/−^ and WT littermates aged 8–10 weeks were administered Lieber-DeCarli EtOH-containing or isocaloric control diets (Products F1258SP and F1259SP, respectively; BioServ, Flemington, NJ, USA) as follows: 1% (*v*/*v*) and 2% (*v*/*v*) for two days each, 5% (*v*/*v*) for 2 weeks, and then 4% (*v*/*v*) for one week. On the final day, the EtOH group received a binge of EtOH (5 g/kg) delivered by oral gavage. Animals were euthanized 9 h after the EtOH binge. There were four experimental groups in this study: WT PF (*n* = 8), WT + EtOH (*n* = 10), *Fpr2*^−/−^ PF (*n* = 5), and *Fpr2*^−/−^ + EtOH (*n* = 8). All animal experiments were conducted in accordance with the guidelines set forth by the University of Louisville Institutional Animal Care and Use Committee under an approved protocol (number 18418 to IAK).

### 2.2. Measurement of Plasma ALT Activity and Endotoxin Levels

Plasma ALT activity was determined using the Infinity^TM^ ALT Reagent (Thermo Fisher Scientific, Waltham, MA, USA). Plasma endotoxin levels were measured with the Chromo LAL kit from Associates of Cape Cod, Inc. (East Falmouth, MA, USA) according to the manufacturer’s instructions.

### 2.3. Immunoassay of TNFα in Liver Tissue

Liver samples (~100 mg) were homogenized in modified RIPA buffer (50 mM Tris, 150 mM NaCl, 0.1% Triton X-100, 0.5% sodium deoxycholate, 0.1% SDS) supplemented with protease and phosphatase inhibitors (Halt™, Thermo Fisher Scientific, Waltham, MA, USA) in tubes filled with 0.5 mm glass beads (BioSpec, Bartlesville, OK, USA). Following centrifugation at 16,000× *g*, supernatants were collected, and protein concentrations were determined by the BCA method. A total of 600 µg of liver protein was analyzed on the V-PLEX immunoassay platform for TNFα. The data were collected on the MESO Sector S 600 instrument and then analyzed using Discovery Workbench v. 4.0 software (all from MesoScale Discovery, Rockville, MD, USA).

### 2.4. Histopathological and Immunohistochemical Analysis of Liver Tissue

Formalin-fixed, paraffin-embedded liver samples were sectioned (5 μm), stained with either H & E, chloroacetate esterase (CAE, Sigma Aldrich, St. Louis, MO, USA), TUNEL (ApopTag, EMD Millipore Sigma, St. Louis, MO, USA), or immunostained for CD163 (Abcam, Cambridge, UK) and PCNA (Cell Signaling Technology, Danvers, MA, USA). Quantification was performed by counting CAE-positive, TUNEL-positive, or PCNA-positive cells in a random series of ten digital images per section (200× field). The number of positive cells was summed and averaged to obtain an estimate for each mouse (*n* = 5–10 mice/group). The cryopreserved liver sections were cut into 5 μm sections and then stained with Oil Red O and counterstained with hematoxylin. The percentage of Oil Red O-positive or CD163-positive area was calculated with Image J. For each mouse liver section, ten randomized images were used, following a previously described method [19].

### 2.5. Liver Triglycerides Extraction and Measurement

Liver lipid extracts were measured as previously described [20] using the Infinity^TM^ Triglyceride Liquid Stable Reagent (Thermo Fisher Scientific, Waltham, MA, USA).

### 2.6. RNA Isolation and Real-Time Quantitative Reverse Transcription PCR (RT-qPCR)

Total RNA from the liver was isolated with Trizol reagent (Thermo Fisher Scientific, Waltham, MA, USA) as described by the manufacturer, and any contaminating genomic DNA was removed by digestion with DNase I (Thermo Fisher Scientific, Waltham, MA, USA). cDNA was synthesized with qScript cDNA Supermix (Quanta Biosciences, Beverly, MA, USA) from 1 μg of RNA. RT-qPCR assays were performed with PerfeCTa SYBR Green Fast Mix (Quanta Biosciences) on the BioRad CFX384 instrument (Bio-Rad, Hercules, CA, USA). The data were analyzed by the ΔΔCt method [21]. Primers for RT-qPCR analysis are listed in Supporting Table S2.

### 2.7. Flow Cytometry Analysis of Neutrophils

Neutrophils were isolated from whole blood acquired from naïve WT and *Fpr2*^−/−^ mice (*n* = 3 per genotype) by immunomagnetic negative selection with the EasyStep Mouse Neutrophil Enrichment Kit (StemCell, Vancouver, CA, USA). Preparations with >95% neutrophils (Ly6G^+^/CD11b^+^) were used for assays. Neutrophils were incubated with or without lipopolysaccharide (LPS) (100 ng/mL) for 30 min, followed by assessment of oxidative burst (i.e., superoxide via MitoSOX staining). Neutrophils were analyzed by flow cytometry using a BD FACS Canto II flow cytometer (BD Biosciences, Franklin Lakes, NJ, USA), and data were analyzed via FlowJo software (Ashland, OR, USA).

### 2.8. Measurement of Liver MPO Activity and H_2_O_2_ Levels

Liver lysates (200 μg of protein) were used to detect MPO activity and H_2_O_2_ levels using the myeloperoxidase chlorination activity assay (Cell Biolabs, San Diego, CA, USA) and a peroxide assay kit (Sigma-Aldrich, St. Louis, MO, USA), respectively.

### 2.9. Flow Cytometry Analysis

BMDMs and hepatic non-parenchymal cells were isolated from WT and *Fpr2*^−/−^ mice (*n* = 3 per genotype) as previously described [22]. Cells were then immunostained with PE-F4/80, FITC-Ly6C, and PerCy5.5-Cd11b (Invitrogen, Waltham, MA, USA) to identify the monocyte (F4/80^lo^, Ly6C^hi^, Cd11b^+^) and MoMF populations (F4/80^hi^, Ly6C^lo^, Cd11b^+^) by flow cytometry.

### 2.10. BMDM and Neutrophil Co-Culture Experiment

Bone marrow was isolated from WT and *Fpr2*^−/−^ (*n* = 3 per genotype) as described in Section 2.9. Neutrophils were isolated from the bone marrow by positive selection using Ly6G antibodies and MACS LS columns (Miltenyi, Gaithersburg, MD, USA). WT and *Fpr2*^−/−^ bone marrow cells and neutrophils were then cross-cultured at a 5:1 ratio (500,000 bone marrow to 100,000 neutrophils) [23,24] in BMDM differentiation media ± catalase (100 nM). The culture medium was changed after 48 h to remove dead and non-adherent cells. The culture was continued in L929, which contained media, for an additional 5 days. Cells were then immunostained and analyzed by flow cytometry as described in Section 2.9.

### 2.11. Statistical Analysis

Data were analyzed using an unpaired Student’s *t*-test (two groups) and a one-way ANOVA (more than two groups), followed by the appropriate post hoc analyses to determine significant differences between experimental groups. Data are presented as the mean ± standard error of the mean (SEM). A *p* < 0.05 was considered statistically significant.

## 3. Results

### 3.1. Fpr2^−/−^ Mice Developed Exacerbated Acute-on-Chronic Alcohol-Induced Liver Injury and Inflammation

WT and *Fpr2*^−/−^ mice were fed either an EtOH or control isocaloric diet (EtOH-fed or pair-fed [PF], respectively) for 4 weeks, followed by a single EtOH binge at the end of the feeding protocol. Both WT and *Fpr2*^−/−^ mice tolerated the experimental protocol, and no mortality was observed. Food consumption was similar for all experimental groups (Table S1). The effects of genotype on metabolic parameters in response to EtOH administration are presented in Table S1. Body weight increase was significantly higher in EtOH-fed mice relative to PF for both WT and *Fpr2^−/−^* mice. The liver-to-body weight ratio was reduced in *Fpr2*^−/−^ mice fed EtOH compared to WT EtOH mice, while the white adipose tissue-to-body weight ratio was only significantly higher in *Fpr2*^−/−^ EtOH mice relative to *Fpr2^−/−^* PF mice.

**Figure 1:**
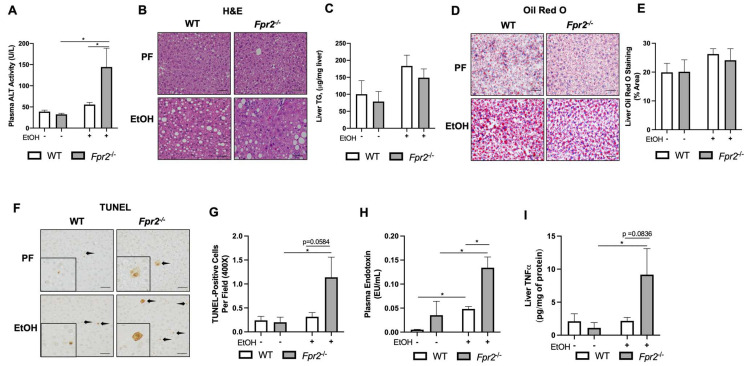
*Fpr2^−/−^* mice developed exacerbated acute-on-chronic alcohol-induced liver injury and inflammation. (**A**) Plasma ALT activity. (**B**) Representative H & E-stained liver sections were imaged at 200×. The scale bar is 100 μm. (**C**) Liver triglyceride measurement. (**D**,**E**) Representative Oil Red O-stained liver sections were imaged at 400× and quantified via Image J to quantify the percent area of Oil Red-O staining. (**F**) Representative TUNEL-stained liver sections were imaged at 400×. The scale bar is 100 μm. (**G**) Quantification of TUNEL-positive cells is from the average of 10 randomized fields per mouse. (**H**) Plasma endotoxin levels. (**I**) Hepatic TNFα levels. Data are presented as mean ± SEM and were statistically compared by a one-way ANOVA with Sidak’s multi-comparison test for parametric data and a Kruskal–Wallis test with Dunn’s multiple comparisons test for non-parametric data; * *p* < 0.05.

*Fpr2* deficiency significantly exacerbated acute-on-chronic alcohol-induced liver injury in EtOH-fed *Fpr2*^−/−^ mice as compared to WT EtOH mice, as determined by elevated plasma ALT activity (Figure 1A). Liver steatosis was modestly increased in both WT and *Fpr2*^−/−^ EtOH-fed mice as determined by liver hematoxylin and eosin (H & E) staining (Figure 1B), liver triglyceride measurement (Figure 1C), and Oil Red O staining (Figure 1D,E) and was comparable between genotypes. The number of TUNEL-positive cells was significantly increased in *Fpr2*^−/−^ EtOH-fed mice but not in WT EtOH-fed mice compared to PF mice, indicating a higher level of EtOH-induced hepatocellular death in the *Fpr2*^−/−^ mice than in the WT mice (Figure 1F,G). Further, EtOH administration resulted in increased endotoxemia (Figure 1H) in both genotypes and was significantly higher in EtOH-fed *Fpr2*^−/−^ vs. WT mice. Compared to PF mice, hepatic TNFα levels, a marker of inflammation, were significantly elevated in *Fpr2*^−/−^ mice fed EtOH but not in WT mice fed EtOH (Figure 1I). Collectively, our data demonstrated that loss of *Fpr2* resulted in more severe EtOH-induced liver injury and inflammation.

### 3.2. Effect of Fpr2 Genetic Ablation on Markers of Liver Regeneration in Response to EtOH

**Figure 2:**
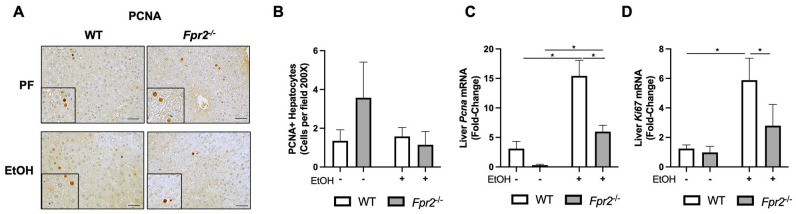
Effect of Fpr2 genetic ablation on markers of liver regeneration in response to EtOH. (**A**,**B**) Representative PCNA-immunostained liver sections were imaged at 400× and quantified. The scale bar is 100 μm. PCNA-positive hepatocytes were manually counted and averaged for 10 randomized fields for each mouse liver section. (**C**,**D**) Hepatic *Pcna* and *Ki67* gene expression. Data are presented as mean ± SEM and were statistically compared by a one-way ANOVA with Sidak’s multi-comparisons test for parametric data and a Kruskal–Wallis test with Dunn’s multiple comparison tests for non-parametric data; * *p* < 0.05.

Compromised liver regeneration is a hallmark of severe ALD [7]. Therefore, we investigated the effect of *Fpr2* deficiency on this process. Immunohistochemical staining revealed no quantitative differences in PCNA-positive hepatocytes among experimental groups (Figure 2A,B). RT-qPCR analysis showed that both WT and *Frp2**^−/−^*** mice had EtOH-induced *Pcna* and *Ki67* gene expression (Figure 2C,D). Of note, the levels of these markers were significantly lower in *Fpr2*^−/−^ vs. WT EtOH-fed animals, suggesting that *Fpr2* genetic ablation may compromise liver regeneration in response to an EtOH insult. There were no statistically significant differences between WT PF and *Fpr2*^−/−^ PF mice in *Pcna* (likely due to the high intragroup variability in the WT PF group) as well as *Ki67* gene expression.

**Figure 3:**
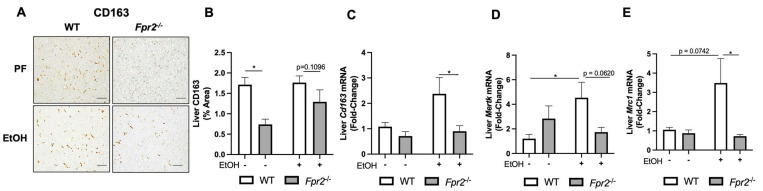
*Fpr2* deletion resulted in reduced hepatic restorative MoMFs in vivo. (**A**,**B**) Representative CD163-immunostained liver sections were imaged at 400× and quantified. The scale bar is 100 μm. Images from 10 randomized fields per mouse liver section were analyzed in Image J to quantify the average percent area of CD163. (**C**) Hepatic *Cd163* gene expression. (**D**,**E**) Hepatic gene expression of *Mertk* and *Mrc1.* Data are presented as mean ± SEM and were statistically compared by a one-way ANOVA with Sidak’s multi-comparisons test for parametric data and a Kruskal–Wallis test with Dunn’s multiple comparisons test for non-parametric data; * *p* < 0.05.

Multiple cell types participate in liver regeneration, including restorative MoMFs [25]. In response to EtOH administration, *Fpr2* genetic deletion resulted in reduced numbers of hepatic MoMFs in *Fpr2*^−/−^ EtOH as compared to WT EtOH-fed mice. This was shown by decreased hepatic staining (Figure 3A,B) and expression of *Cd163* (Figure 3C), *Mertk* (Figure 3D), and *Mrc1* (Figure 3E), well-recognized MoMFs markers [25,26,27]. Flow cytometry analysis further confirmed that *Fpr2*^−/−^ mice had intrinsically lower levels of hepatic MoMFs as compared to WT mice (Figure S2).

**Figure 4:**
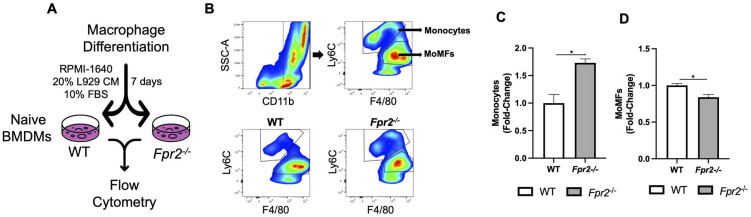
*Fpr2^−/−^* bone marrow progenitors had reduced MoMF differentiation potential in vitro. (**A**) Model of BMDM differentiation. (**B**) Gating strategy for flow cytometry analysis, and representative flow cytometry plots for WT and *Fpr2*^−/−^ monocytes and MoMFs. (**C**,**D**) Quantification of monocytes and MoMFs for WT and *Fpr2*^−/−^ cells. The data for this figure are an average of three independent experiments using individual mice of each genotype. Data are presented as mean ± SEM and were statistically compared by an unpaired Student’s *t*-test; * *p* < 0.05.

Next, we examined the role of *Fpr2* in MoMF differentiation in vitro using bone marrow-derived macrophages (BMDMs) isolated from naïve WT and *Fpr2*^−/−^ mice. A standard macrophage differentiation protocol [28] was followed by flow cytometry analysis to measure the number of monocytes and MoMFs (Figure 4A). We found that the monocyte population was significantly higher, while the MoMF population was significantly lower in *Fpr2*^−/−^ relative to WT mice-derived cells (Figure 4B–D). This suggests that loss of *Fpr2* expression compromises monocyte-to-macrophage differentiation potential.

### 3.3. Liver Damage in EtOH-fed Fpr2**^−/−^** Mice was Associated with Alterations in Neutrophil Oxidative Burst

**Figure 5:**
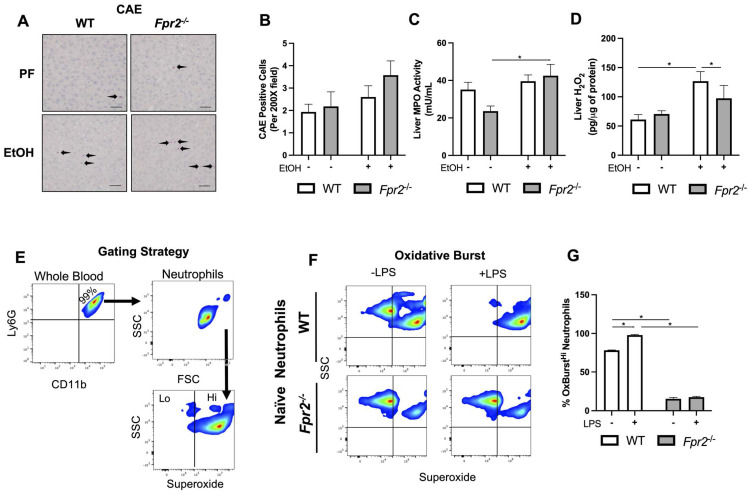
Liver damage in EtOH-fed *Fpr2^−/−^* mice was associated with compromised neutrophil oxidative burst. (**A**) Representative CAE-stained liver sections were imaged at 400×. Scale bar is 100 μm (**B**) Quantification of CAE-positive cells from 10 randomized fields of view were averaged per mouse. (**C**) Hepatic MPO activity. (**D**) Hepatic H_2_O_2_ levels. (**E**) Flow cytometry gating strategy for assessment of oxidative burst in neutrophils. (**F**) Representative flow cytometry plots of WT and *Fpr2*^−/−^ neutrophil assessment for oxidative burst. (**G**) Quantification of high oxidative burst (increased superoxide) neutrophil populations between genotypes. Data for (**E**–**G**) are an average of three independent experiments using individual mice of each genotype. Data are presented as mean ± SEM and were statistically compared by a one-way ANOVA with Sidak’s multi-comparisons test for parametric data and a Kruskal–Wallis test with Dunn’s multiple comparisons test for non-parametric data; * *p* < 0.05.

It has been recently shown that hepatic neutrophils may promote monocyte differentiation to MoMFs via the release of oxidative species (e.g., H_2_O_2_ and O_2_^−^) [29]. First, we examined the effects of genotype and EtOH administration on hepatic neutrophil infiltration. There was a trending increase in hepatic neutrophils in response to EtOH in both genotypes (although not significant), which was higher in *Fpr2*^−/−^ mice (Figure 5A,B). Another marker of neutrophils, hepatic MPO activity, was significantly elevated by EtOH in *Fpr2*^−/−^ but not in WT mice (Figure 5C). Next, hepatic H_2_O_2_ levels were examined, which were increased in EtOH-fed mice in both genotypes, reaching statistical significance, however, only in WT animals (Figure 5D). Importantly, there was a significant decrease in hepatic H_2_O_2_ levels in EtOH-fed *Fpr2*^−/−^ vs. WT mice. Since neutrophil oxidative burst is compromised in severe ALD [30] and can positively regulate MoMF differentiation [29], we further investigated the effects of *Fpr2* ablation on neutrophil oxidative burst in vitro. Both basal and LPS-stimulated oxidative bursts, as measured by superoxide production, significantly decreased in *Fpr2*^−/−^ neutrophils (Figure 5E,F). These findings may indicate that neutrophil-derived factors contributing to MoMF differentiation are altered with *Fpr2* ablation.

### 3.4. Fpr2^−/−^ Bone Marrow Cells can Differentiate into MoMFs when Co-Cultured with WT Neutrophils

**Figure 6:**
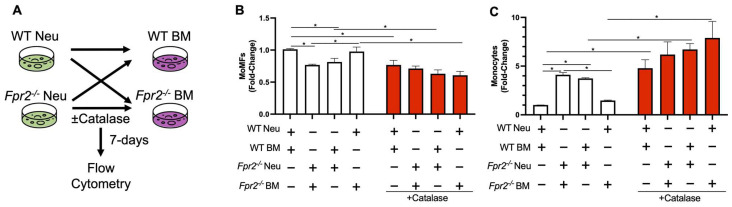
*Fpr2^−/−^* bone marrow cells can differentiate into MoMFs when co-cultured with WT neutrophils. (**A**) Schematic of WT and *Fpr2*^−/−^ neutrophils and bone marrow cells being co-cultured and MoMF differentiation being evaluated by flow cytometry analysis. Changes in (**B**) MoMFs and (**C**) monocytes under various co-culture conditions. The data for this figure are an average of three independent experiments using individual mice of each genotype. Data are presented as mean ± SEM and were statistically compared by a one-way ANOVA with Sidak’s multi-comparisons test for parametric data and a Kruskal–Wallis test, * *p* < 0.05.

Recently, it has been demonstrated that neutrophils can promote MoMF differentiation via an H_2_O_2_-dependent mechanism [29]. To test if a reduction in H_2_O_2_ production by *Fpr2*^−/−^ neutrophils contributed to compromised MoMF differentiation, we co-cultured neutrophils and bone marrow cells isolated from WT and *Fpr2*^−/−^ mice (Figure 6A). When neutrophils and BM cells from *Fpr2*^−/−^ mice were cultured, there were reduced numbers of MoMFs as compared to when cells from WT mice were cultured (Figure 6B). Co-culture of WT BM cells with *Fpr2*^−/−^ neutrophils resulted in a similar reduction in MoMFs. Next, when WT neutrophils and *Fpr2*^−/−^ BM cells were co-cultured, there was a rescue in the number of MoMFs equal to that observed when WT neutrophils and BM cells were co-cultured. There was a corresponding increase or decrease in monocytes observed for the various treatments, as seen in Figure 6C. Lastly, these effects appear to be due to H_2_O_2_ since catalase-treated cells had a reduction in MoMFs with a concomitant increase in monocytes, as previously reported [29,31].

## 4. Discussion

In the present study, we investigated the effects of the genetic deletion of *Fpr2* in an experimental animal model of ALD. FPR2 is a G-protein-coupled receptor that is highly expressed in multiple immune cell types [32,33] and interacts with numerous ligands to regulate distinct signaling cascades and processes, including immune responses [10,34]. We found that loss of *Fpr2* exacerbated experimental EtOH-induced liver injury and inflammation, which was associated with compromised liver regeneration. Our findings are consistent with recent studies demonstrating worsened pathology in *Fpr2*^−/−^ mice in animal models of various diseases. For example, compared to WT, female *Fpr2*^−/−^ mice in a dietary model of non-alcohol-associated fatty liver disease had enhanced hepatocellular death and inflammation, as shown by elevated hepatic TNFα levels [15]. In a separate study, LPS-challenged *Fpr2*^−/−^ mice had increased liver injury, elevated levels of hepatic TNFα and immune cell infiltration, and reduced liver regeneration [16]. Our observations, paired with previously reported studies, demonstrate a shared phenotype of exacerbated liver injury, inflammation, and moderately compromised regeneration in *Fpr2*^−/−^ mice following distinct insults such as diet, LPS, or alcohol.

Compromised liver regeneration is frequently observed in patients with ALD [7,8,35]. However, underlying mechanisms are still under-investigated and not fully understood. It has been shown that the depletion of hepatic macrophages results in compromised liver regeneration in EtOH-fed mice [36]. Recently, restorative MoMFs have been identified as an important cell population in the coordination of liver regeneration via the resolution of inflammation and the release of pro-regenerative growth factors [25]. In our animal model, we observed alterations in markers of liver regeneration in response to EtOH in *Fpr2*^−/−^ vs. WT mice. We also found reduced hepatic MoMF markers in *Fpr2*^−/−^ mice in vivo and reduced MoMF differentiation capacity in vitro in BMDMs from *Fpr2*^−/−^ animals. This suggests that FPR2 may play a role in MoMF differentiation via interaction with one of its ligands. Previously, FPR2 activation by a synthetic FPR2 agonist was shown to increase the number of pro-resolution macrophages in an animal model of myocardial infarction [37]. Since many ligands interact with FPR2, future studies are warranted to determine which FPR2 ligands may promote monocyte-to-MoMF differentiation. Other MoMF factors have also been described, such as IL-10 [38] and SLPI [27]. For example, treatment with blocking antibodies to IL-10 delayed the monocyte-to-MoMF transition in an animal model of liver injury [38]. Previously, *Fpr2*^−/−^ mice have been shown to produce less IL-10 [12], suggesting that loss of *Fpr2* may impact MoMF differentiation via reduced IL-10 production.

Monocytes can differentiate into restorative MoMFs by neutrophil-derived signals as well, such as reactive oxygen species (ROS), that in turn stimulate hepatocyte proliferation [29]. In our study, we observed increased hepatic neutrophil infiltration but compromised function, including oxidative burst, associated with the depletion of neutrophil-derived ROS (e.g., H_2_O_2_ and superoxide) in *Fpr2*^−/−^ mice. Likely, a loss of neutrophil ROS contributed to the reduced number of MoMFs observed in *Fpr2*^−/−^ mice. Indeed, we found that WT neutrophils could restore *Fpr2*^−/−^ MoMF differentiation, which was attenuated by catalase. Of note, monocytes can also produce H_2_O_2_ upon treatment with M-CSF (present in L929 media), which can contribute to their differentiation [31], thus explaining why catalase treatment did not fully ablate MoMF differentiation in the present study. The role of FPR2 in neutrophil oxidative burst was also previously demonstrated in in vitro studies. This shows that FPR2 activation in HL-60 cells (a neutrophil-like cell line) by the FPR2 ligand BMS-986235 stimulated an oxidative burst, but this was completely ablated with the loss of FPR2 [37]. Phosphoregulation of NADPH oxidase subunits was recently identified as one of the mechanisms by which FPR2 may mediate an oxidative burst [39]. Our findings are relevant to human ALD since AH patients have impaired neutrophil oxidative burst [30], which may contribute to altered liver regeneration. Future studies will aim to investigate the neutrophil and MoMF crosstalk in pre-clinical and clinical ALD and determine if FPR2 agonists have therapeutic potential.

The current study is novel and relevant to the field of ALD, but it has some potential limitations. In this study, only female mice were examined, taking into account that females develop exacerbated ALD when compared to males [40,41]. However, sex-specific differences are important considerations for future studies. The use of immune cells from the bone marrow and blood for in vitro studies might be another limitation of this study. However, neutrophils and monocytes originate from the bone marrow and infiltrate the liver upon chemokine signaling in response to liver injury [42], suggesting that the effects observed in these studies may mimic what occurs in the liver. Lastly, since *Fpr2* is also expressed in hepatocytes [33], future studies are warranted to examine its hepatocyte cell-specific effects in ALD.

## 5. Conclusions

*Fpr2*^−/−^ mice developed exacerbated alcohol-induced liver injury and inflammation in an experimental model of ALD. Additionally, the limited liver regeneration observed in *Fpr2*^−/−^ mice in response to EtOH is due to mechanisms involved in immune cell dysregulation, such as a reduced number of MoMFs, which is likely a result of impaired differentiation due to reduced neutrophil-derived H_2_O_2_. This study suggests that FPR2 may play a pivotal role in the differentiation of monocytes into MoMFs in ALD. The observed phenotype in *Fpr2*^−/−^ mice is reflective of what occurs in human AH, providing a rationale for evaluating FPR2 as a therapeutic target in human ALD.

## Data Availability

The data presented in this study are available within this article and supplementary material.

## Supplementary Materials

The following supporting information can be downloaded at: https://www.mdpi.com/article/10.3390/biology12050639/s1, Figure S1: Liver *Fpr2* mRNA expression; Figure S2: Liver MoMFs; Table S1: Metabolic characteristics of experimental mice. Table S2: qPCR Primers.
