# Supplementary material for: Fpr2−/− Mice Developed Exacerbated Alcohol-Associated Liver Disease
Source: Biology (Basel). 2023 Apr 23;12(5):639. doi: 10.3390/biology12050639 (PMC10215685; doi:10.3390/biology12050639)
Supplement: Supplementary file 1 [file biology-12-00639-s001.zip › biology-2235164-supplementary.pdf]

Supplemental Materials

Fpr2$^{-/-}$ mice developed exacerbated alcohol-associated liver disease

Josiah E. Hardesty [1], Jeffrey B. Warner [1,2], Ying L. Song [1], Alison Floyd[1], Craig J. McClain[1,2,3,4,5], Dennis R. Warner[1] and Irina A. Kirpich [1,2,4,5,6*]

Supplemental Table S1: Metabolic Characteristics of Experimental Mice

	**WT PF (n=8)**	***Fpr2***$^{-/-}$ **PF (n=5)**	**WT EtOH (n=10)**	***Fpr2***$^{-/-}$ **EtOH (n=8)**
**Initial BW, g**	21.83 ± 1.21	18.18 ± 0.48 [a]	18.05 ± 0.24 [a]	18.39 ± 0.56
**Final BW, g**	26.20 ± 1.53	21.60 ± 0.77 [a]	22.85 ± 0.44 [a]	22.89 ± 0.83
**Liver/BW, %**	3.50 ± 0.15	3.99 ± 0.11 [a]	4.29 ± 0.07 [a]	3.66 ± 0.17 [c]
**WAT/BW, %**	2.32 ± 0.43	1.49 ± 0.24	2.16 ± 0.2	2.64 ± 0.25 [b]
**Food Consumption, mL/day per mouse**	*	*	13.63 ± 0.32	13.14 ± 0.50

Abbreviations: BW: body weight, WAT: white adipose tissue. * denotes that PF mice consumed the same amount of diet as their EtOH-fed counterparts. Statistical significance and comparison are denoted by the following letters: [a]: *vs* WT PF, [b]: *vs Fpr2*$^{-/-}$ PF; [c]: *vs* WT EtOH. One-way ANOVA, $p < 0.05$.

Supplemental Table S2: qPCR Primers

**Gene**	**Forward Primer Sequence**	**Reverse Primer Sequence**
18s	CTCAACACGGGAAACCTCAC	CGCTCCACCAACTAAGAACG
Pcna	TGTGCCCCTTGTTGTAGAGT	AAAGACCTCAGGACACGCTG
Ki67	TCACCTGGTCACCATCAAGC	TCAATACTCCTTCCAAACAGGCA
Cd163	GTGCTGGATCTCCTGGTTGT	CGTTAGTGACAGCAGAGGCA
Mertk	GCGAGTTTGGGACGTTGGTG	ACTTCTCGGCAGTGCTCC
Mrc1	AGGGACCTGGATGGATGACA	AGGGAAGGGTCAGTCTGTGT
Fpr2	CACAGGAACCGAAGAGTGTAAG	CACCATTGAGAGGATCCACAG

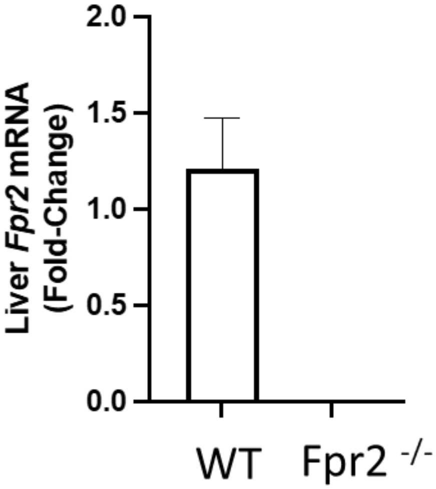

Supplemental Figure S1. Liver *Fpr2* mRNA expression in WT and *Fpr2*$^{-/-}$ mice. Data are presented as mean ± SEM.

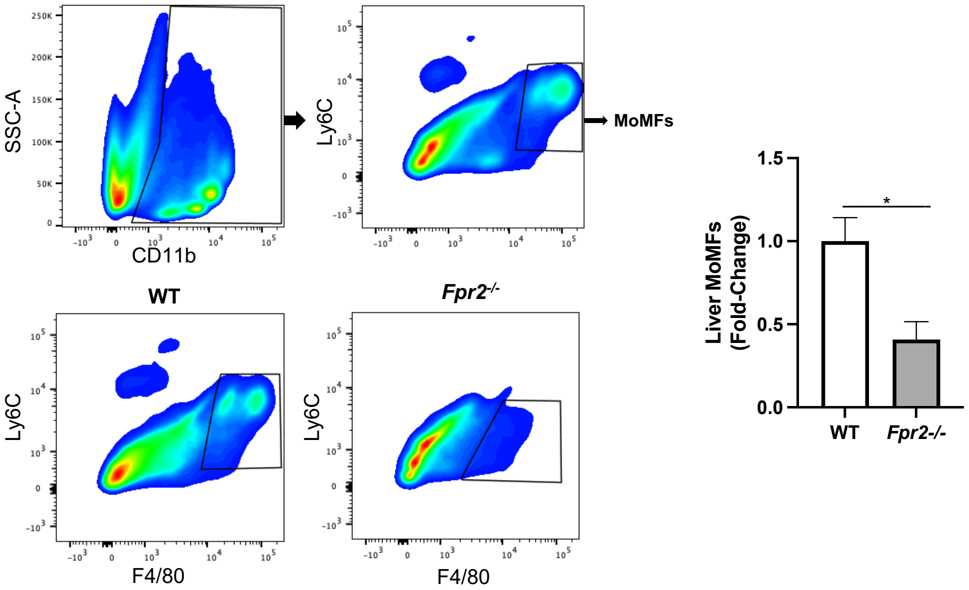

Supplemental Figure S2. Gating strategy to measure hepatic MoMFs and the associated quantification in WT and *Fpr2*$^{-/-}$ mice. Data are presented as mean ± SEM. The data were acquired from independent experiments using 3 individual mice from each genotype.
